# Towards a Unified European View of Clinical Evidence: What ‘Health Technology Assessment Organizations’ Can Learn from Regulatory Experience

**DOI:** 10.3390/jmahp13020019

**Published:** 2025-04-28

**Authors:** Karl Broich, Wiebke Löbker

**Affiliations:** Federal Institute for Drugs and Medical Devices (BfArM), 53175 Bonn, Germany; wiebke.loebker@bfarm.de

**Keywords:** EU-HTA, health technology, national competent authority, EMA, centralized marketing authorization, benefit–risk assessment

## Abstract

The harmonization of pharmaceutical regulations within the European Union has been a crucial step towards ensuring high safety standards and efficient access to innovative medicines. The evolution from fragmented national regulations to a unified legal framework has streamlined the marketing authorization process, fostered scientific collaboration, and reduced administrative burdens. The establishment of the centralized marketing authorization procedure and the European Medicines Agency (EMA) has played a pivotal role in coordinating regulatory efforts across member states. This article examines the historical developments, regulatory milestones, and the impact of harmonization on pharmaceutical assessments. Furthermore, it explores key lessons learned—including the value of centralized coordination, standardization, capacity and knowledge-sharing, transparency and trust—from the regulatory landscape that could inform the evolving Health Technology Assessment (HTA) framework in the EU.

## 1. Introduction

With the EU regulation “on the assessment of health technologies”, the European Commission introduced a single Health Technology Assessment (HTA) for medicinal products across Europe from January 2025 onwards [1]. According to the regulation [1], while the results of the joint clinical assessment are to be considered for national assessments by the member states, the evaluation of economic aspects, pricing, and reimbursement decisions will remain under national jurisdiction. This initiative by the European Commission has sparked discussions in many countries where HTA procedures—such as the early benefit assessment in Germany—are already well-established. These harmonization efforts towards a centralized clinical assessment have raised concerns about the potential dilution of established (health quality) standards [2]. The processes and criteria for assessing clinical evidence in the context of the benefit–risk assessment of medicinal products during the approval process have also undergone significant harmonization. Starting from a heterogeneous legal framework and diverse approaches in the European countries, numerous modifications through the centralized approval procedure have led to substantial alignment, and are currently under evaluation by the pharmaceutical legislation revision initiative [3]. This process of a centralized, harmonized assessment began in the mid-1960s with Directive 65/65/EEC. This article summarizes experiences and lessons learned by the regulatory authorities along this gradual path toward a unified European perspective on clinical evidence and towards a European Medicines Regulatory Network, and how the current collaborative system between the European Medicines Agency (EMA) and the national European regulatory authorities was established and continuously further developed.

## 2. Centralized Single Marketing Authorization: Experience from the Development of Harmonized Standards and Harmonized Assessments of Clinical Evidence

Before starting the harmonization efforts in Europe, the legal framework and thus the conditions for the marketing of medicinal products were very heterogeneous. In the course of the development of a joint European legal framework to reduce or eliminate obstacles in the internal market, the pharmaceutical sector has also experienced a gradual harmonization and optimization of the legal framework and the (evaluation) processes for the manufacture and authorization for the distribution of medicinal products—with the aim of achieving a uniformly high level of protection of public health, particularly through rapid access to innovative and safe medicinal products, as well as more stringent control of the safety of medicinal products [4].

One of the central starting points for the pharmaceutical sector in this context is the Rome Treaties on the harmonization of European legislation, which, amongst others, called for a national pharmaceutical law. Unlike the other members of the European Economic Community, Germany did not have its own pharmaceutical law at that time. In November 1961, the Federal Government of the time fulfilled this obligation and established a Ministry of Health; in the same year, the first Medicines Act came into force in Germany. While this first version of the Medicinal Products Act did not yet provide for an obligation to evaluate the efficacy and safety of a medicinal product, but only for registration, numerous amendments and guidelines came into force in the following years, which were mainly introduced to align the country-specific legal requirements with European law [5].

Another important milestone for the harmonization of legal provisions in the EU regarding medicinal products was laid down with Directive 65/65/EEC. This directive contained important definitions, some of which were used for the first time, such as the term ‘medicinal product’, as well as requirements and conditions for a marketing authorization for medicinal products: proof of quality, therapeutic efficacy, and safety [4]. With the Directives 75/318/EEC and 75/319/EEC, the regulations for the supervision of medicinal products were gradually harmonized further. Not only were minimum requirements for manufacturing and testing and details for manufacturing and import licences laid down, but also a Committee for Proprietary Medicinal Products—with representatives of the member states and the Commission—was set up to facilitate the granting of marketing authorizations for the same pharmaceutical specialty in several member states, i.e., to avoid the need for a new review of a medicinal product that has already been authorized in one member state to avoid additional work in another member state (Mutual Recognition Procedure, MRP).

Another important milestone for the harmonization of legal provisions in the EU regarding medicinal products was laid down with Directive 65/65/EEC. This directive contained important definitions, some of which were used for the first time, such as the term ‘medicinal product’, as well as requirements and conditions for a marketing authorization for medicinal products: proof of quality, therapeutic efficacy, and safety [4]. With the Directives 75/318/EEC and 75/319/EEC, the regulations for the supervision of medicinal products were gradually harmonized further. Not only were minimum requirements for manufacturing and testing and details for manufacturing and import licences laid down, but also a Committee for Proprietary Medicinal Products—with representatives of the member states and the Commission—was set up to facilitate the granting of marketing authorizations for the same pharmaceutical specialty in several member states, i.e., to avoid the need for a new review of a medicinal product that has already been authorized in one member state to avoid additional work in another member state (Mutual Recognition Procedure, MRP).

These guidelines were implemented in Germany with the revision of the Medicinal Product Act of 1976, with the introduction of a binding marketing authorization procedure with the requirement to provide proof of quality, efficacy, and safety.

The introduction of a centralized marketing authorization procedure by Regulation (EEC) No. 2309/93 of 22 July 1993 represented a further major milestone. These regulations for a centralized approval procedure, initially applied to technologically advanced medicinal products (from biotechnology), followed the goal of ensuring a common Europe-wide assessment of these complex products with the best available expertise from the EU member states—and thus to prevent such innovative products from not being sufficiently tested or not being tested in an appropriate time frame due to a lack of expertise in one or more member states, and thus from not being able to be placed on the market.

Overall, the submission of a single application for marketing authorization, in addition to the pooling of the best available scientific expertise from the European member states for a benefit–risk assessment, based on consistently harmonized standards, pursued the goal of ensuring equivalent patient safety standards in Europe. In parallel, it also enabled a focus on the essential scientific core questions regarding the therapeutic benefit, quality, and safety of a medicinal product by further reducing the administrative burden.

In parallel to the centralized approval procedure, the European Agency for the Evaluation of Medicinal Products (EMEA; nowadays European Medicines Agency (EMA)), was also established by this directive in 1995. Since then, the EMA, through its secretariat and scientific committees consisting of representatives from the European (and EEA) member states, has played a central coordinating role in the authorization and monitoring of innovative medicinal products in the EU.

In the same year, the first marketing authorization for a medicinal product (Gonal-F (follitropin alfa)) was granted under the centralized marketing authorization procedure [6].

Based on a report to be prepared by the Commission to evaluate the single European marketing authorization procedures, further fundamental adjustments to the legal framework and the benefit–risk assessment procedures were carried out with Directive 2001/83/EC and, in particular, Regulation (EC) No 726/2004.

Directive 2001/83/EC consolidated all directives applicable at that time to medicinal products for human use into a single directive. This directive, which has been continuously modified and adopted by further regulations and directives, codifies the principles for the manufacture, marketing, and monitoring of medicinal products. With the adoption of the directive, the introduction of the decentralized procedure (DCP) also created a further possibility within the EU for obtaining national marketing authorizations for medicinal products in several member states at the same time.

Regulation (EC) No 726/2004 ultimately replaced Regulation (EEC) 2309/93 and provided an extension of the medicinal products for which the central marketing authorization procedure is mandatory. The Regulation also introduced the possibility of a conditional marketing authorization, i.e., a provisional marketing authorization under defined conditions. This form of authorization is considered for medicinal products intended for serious or life-threatening diseases, for which there are currently no effective therapeutic options licensed, and for which the available data show that the benefit–risk ratio is positive and a significant benefit for patients is apparent. This conditional marketing authorization is reassessed annually based on further robust evidence (from further clinical trials e.g., ongoing at the time of authorization, or today also from so-called Real-World Evidence).

Furthermore, the publication of the assessment reports, the European Public Assessment Reports (EPAR), was established to increase the transparency of the decisions [7].

To increase awareness for special conditions and patient groups and to combine needed expertise, new committees were also set up at the EMA—such as the Committee for Orphan Medicinal Products (COMP), which was established in 2000 based on Regulation (EC) No. 141/2000 to increase awareness for rare diseases. Six years later, the basis of Regulation (EC) 1901/2006, the Paediatric Committee (PDCO), was introduced at the EMA to improve the supply of medicinal products for children.

With the implementation of the Pharmacovigilance Risk Assessment Committee (PRAC) in 2012, the pan-European monitoring of drug safety in Europe was further intensified.

In addition to these legal framework conditions, the support options for the development of innovative medicinal products by the EMA and the European medicines regulatory network have also been further developed: in 2010, the EMA and the previously voluntary HTA institutions network, in the European Network for Health Technology Assessment, EUnetHTA [8], joined together, which, among other things, led to better understanding and the establishment of a permanent platform for joint consultation on regulatory and HTA aspects (‘parallel consultation’) [9].

With the PRIME (PRIority MEdicines) initiative, introduced in 2016, the EMA and the European medicines regulatory network support the development of medicinal products for which there is an unmet medical need. This voluntary initiative is based on early, closer interaction with developers of promising innovative medicines, to optimize the development plans and to speed up the evaluation time, to ensure accordance with the current state of the science and guidelines, and thus to reach patients without unnecessary delay [10]. In parallel, Innovation offices have been set up in numerous national regulatory authorities working together in the EU Innovation Network to foster support for innovative approaches [11].

## 3. The European Medicines Regulatory Network—Close Cooperation and Work Sharing Between the National Regulatory Authorities and EMA

The harmonization of requirements in the European Union (EU) for the pharmaceutical sector, the work sharing in the authorization and risk assessment procedures, and in particular the introduction of the centralized marketing authorization procedure and the establishment of the EMA have gradually led to the development of a strong European regulatory network over the past few years. This network can draw on the best available expertise from the individual European member states and thus pursues an efficient approach. The EMA is the coordinating center. Experts are appointed from ~50 national regulatory authorities in the EU and the European Economic Area (EEA) to the EMA’s seven committees and (temporary) working groups, which are currently responsible for scientific evaluation. The Committee for Human Medicinal Products (CHMP) and the Committee for Veterinary Medicinal Products (CVMP) play a key role here, working closely with the other committees (PRAC, COMP, PDCO; Committee on Herbal Medicinal Products (HMPC), Committee for Advanced Therapies (CAT)) and the specific working groups. This structure not only ensures the best available expertise from the European member states, ensuring a high level of procedural efficiency, but also contradicts initial criticisms and concerns regarding the centralized assessment resulting in a decline in national decision-making sovereignty.

The EMA committees, on which (at least) one expert from each member state is usually represented and which are supported by the EMA secretariat, not only undertake the technical assessment of the centrally submitted marketing authorization applications and Europe-wide risk assessment procedures, but also the development and updating of scientific guidelines. These guidelines provide a harmonized interpretation and uniform assessment standards when interpreting and applying the requirements for demonstrating quality, efficacy, and safety as set out in the Community guidelines. Any questions going beyond this are discussed by the Scientific Advice Working Party, the relevant working group of the CHMP, in the context of advice procedures at the national level or at the EMA (Scientific Advice; Protocol Assistance).

For each scientific assessment of a procedure, one of the committee members is appointed as rapporteur or co-rapporteur. The (co-)rapporteur is selected based on objective criteria (e.g., professional expertise, experience in the evaluation of similar procedures/products). The (co-)rapporteur is responsible for the scientific evaluation and for preparing the assessment report (EPAR). Committee decisions should be reached by consensus wherever possible. If unanimity cannot be achieved, the scientific opinion is adopted if an absolute majority of the committee members vote in favor; dissenting positions and representatives of these positions are transparently presented in the EPAR [12]. The scientific assessment report prepared by the rapporteur and co-rapporteur and adopted by the CHMP is the basis for the marketing authorization decision, which is ultimately taken by the European Commission.

## 4. Joint Activities—Regulatory Authorities and HTA Institutions

While the requirements of the regulatory authorities in Europe and thus also for Germany have been very strongly harmonized in recent decades, as described above, this does not yet apply to the HTA and reimbursement decision requirements in Europe. HTA assessments and decisions on reimbursement are currently made at a purely national level; the systems in the member states are very heterogeneous in terms of methodology, value frameworks (e.g., the role of economic evaluation in decision-making), and timing/time frame of the assessment.

In Germany, for example, with the Act on the Reform of the Medicinal Products Market (AMNOG), an early benefit assessment was introduced in 2011 for new medicinal products in accordance with section 35a of the German Social Code, Book V (SGB V). This assessment forms the basis for decisions on the amount to be reimbursed between the manufacturer and the National Association of Statutory Health Insurance Funds (GKV-SV). While the focus of the marketing authorization procedure is on assessing whether a drug is of sufficient quality, efficacy, and safety, the early benefit assessment examines the question of what added therapeutic value (‘additional benefit’) a new drug has compared to the standard of care in Germany and thus why it justifies a higher price. Even though the benefit–risk assessment and the early benefit assessment according to Germany Social Code Book V thus pursue different questions and are based on different assessment criteria, the evaluations and decisions are nevertheless largely based on the same evidence. The challenge for pharmaceutical companies is therefore to design clinical studies that meet the requirements of the regulatory authorities in multinational clinical trials, but at the same time also meet the requirements for the additional benefit assessment in Germany. Since other countries have also implemented complex HTA procedures in some cases, these multinational studies must also meet their requirements, which makes conducting such study designs very complex overall. For companies operating primarily internationally, this means that the market access departments, which are responsible within the company for reimbursement negotiations in the individual countries, must be involved in global development programs much earlier to cover as many requirements as possible.

In this context, scientific advice from the institutions involved is becoming increasingly important in the planning of pivotal studies. At the national level, amongst others in Germany, mutual participation in advisory discussions at the regulatory authorities (here Federal Institute for Drugs and Medical Devices, BfArM and Paul-Ehrlich-Institute, PEI) and at the Federal Joint Committee (G-BA), but above all the participation of the higher federal authorities in the G-BA’s advice according to section 35a Social Code Book V, have become routine in Germany [13]. The offer of joint advice should be used by pharmaceutical companies to obtain information at an early stage about both the requirements for the assessment of the added benefit of drugs according to § 35a SGB V and the requirements for marketing authorization.

This will be more important from January 2025 onwards, when the EU HTA process begins in parallel to the regulatory assessment by the EMA [1]. HTA bodies and the EMA are currently preparing for joint consultations [8].

The accelerated and especially the conditional approval procedures are also an area of conflict between the approval and the assessment of added benefit. These procedures—in which approval is initially granted for a limited period under defined conditions—are often criticized for the fact that at the time of approval (and thus due to the close temporal connection also at the time of the additional benefit assessment), the available evidence is increasingly insufficient to definitively assess the efficacy and safety of these drugs [14]. The digitalization of the healthcare system, the closer networking of the stakeholders, and the continuously increasing amount of data from different sources (‘big data’), which are leading to a change in the pharmaceutical market, will also have an impact on the evaluation processes in the context of marketing authorization and HTA evaluation.

In this context, closer cooperation with early exchange between regulatory authorities and HTA institutions, despite the different tasks and requirements, is of great importance to be able to design clinical trials as efficiently as possible, with a view to the data basis required for both approval and benefit assessment.

Overall, and with a view to the potential introduction of a standardized European-wide HTA joint clinical assessment procedure, which follows immediately after the benefit–risk assessment, further points arise in asking for a close, continuous dialogue and exchange between all stakeholders.

## 5. What Can the EU HTA Landscape Learn from the Road to Harmonized, Central Approval and the EU Medicines Regulatory Network?

The HTA landscape can draw several valuable lessons from the establishment of the EU regulatory medicines network and the centralized assessment procedures of innovative drugs. These lessons are detailed below.

### 5.1. Centralized Coordination with Decentralized Implementation

The EMA functions as a scientific secretariat for coordinating assessments and leveraging expertise from national agencies. Similarly, a successful EU HTA system can benefit from a centralized framework for clinical assessment while respecting national autonomy for economic evaluations and reimbursement decisions. The structure of the scientific committees at the EMA has proven particularly successful in this regard. These committees are composed of experts from the national regulatory authorities or from the European member states/EEA, guaranteeing the best available scientific expertise for the benefit–risk assessments. In addition, increasing transparency, e.g., by publishing the assessment reports (including a presentation of discrepant positions and discussions in the committees) and the guidelines, which usually undergo a public consultation phase, is an important factor that should also be included from the outset in the establishment of a standardized HTA assessment process and the structures required.

### 5.2. Trust-Building via Collaboration

The establishment of mutual trust between the stakeholders in the EU medicines regulatory network was achieved by consistent collaboration, transparent processes, and respect for national expertise. This was achieved amongst others by joint trainings for the medicines regulatory network, mainly by the network itself (EU Network Training Centre EU NTC), common curricula, and regular sessions on lessons learned. HTA organizations can build on this success story by fostering a similar cooperative environment allowing all member states a valuable contribution, and by respecting national sovereignty and conditions.

### 5.3. Standardized Methodologies

The EU regulatory network has developed robust standards and procedures for benefit–risk evaluations, ensuring consistency and the highest quality across EU member states. EU HTA can adopt similar methodologies for the joint HTA clinical assessments, ensuring a common understanding of evidence without compromising national interpretations for economic/reimbursement contexts. In the EMA committees, a common understanding of the regulatory and procedural requirements, and a common set of standards—scientific guidelines—have been or are continuously being developed. This not only enables a harmonized assessment and efficient work sharing, but also ensures that national conditions are taken into account, while at the same time offering developers important assistance in preparing applications. During the EUnetHTA activities, HTA institutions gained experiences in consultation of joint guidelines and standards.

### 5.4. High-Quality Evidence

The regulatory system prioritizes solid clinical evidence and often harmonizes different national approaches or jointly adapts requirements to the current state of scientific knowledge in order to maintain high standards and the evidence requirements (e.g., addressing the integration of real-world evidence into decision-making processes). HTA can learn here by integrating/aligning evidence requirements across the EU member states wherever possible, emphasizing high-quality data for clinical assessments, while allowing flexibility for local health system priorities in parallel and adapting the standards by discussing up-to-date scientific knowledge.

### 5.5. Capacity-Building and Knowledge-Sharing

The EU medicines network emphasizes capacity-building through knowledge-sharing, training programs (EU Network Training Centre), and scientific advice. HTA organizations can establish similar mechanisms to strengthen scientific and technical capacity across member states and enhance mutual learning.

### 5.6. Stepwise Approach and Iterative Optimization

The EU regulatory network evolved through iterative adjustments and amendments over decades, reflecting on challenges and adapting solutions. HTA should anticipate and embrace a phased implementation of harmonized processes, ensuring mechanisms for feedback and continuous improvement. Furthermore, a reflection following experiences and an evaluation, as already foreseen by Article 31 of the EU HTA Regulation after three years, is of great value as the evaluation and the resulting adjustments to the central marketing authorization procedure have clearly shown.

### 5.7. Patient-Centricity and Transparent Decision-Making

The medicines regulatory landscape integrates patient perspectives into regulatory decisions and ensures transparency in decision-making. HTA can similarly enhance patient and stakeholder engagement, building broader acceptance and trust in the assessments.

### 5.8. In the End: Efficiency Gains Through Centralization

Centralized medicinal product assessments reduce the duplication of effort across EU member states, building efficiencies. HTA can aim for similar efficiency in clinical evaluations by pooling resources for joint assessments, allowing national bodies to focus on regional economic and policy considerations.

By learning from the regulatory medicines network’s successes and challenges, the EU HTA system can evolve into a robust, cooperative framework that enhances the quality, efficiency, and impact of health technology assessments across Europe—for the good of patients.

## 6. Discussion

The centralized marketing authorization procedure has demonstrated several advantages, including increased efficiency, improved scientific assessment, and faster access to innovative medicines. By consolidating expertise from national regulatory authorities, the EMA has ensured a high standard of benefit–risk evaluation, mitigating disparities in national assessments. Furthermore, the transparency introduced through European Public Assessment Reports (EPARs) has enhanced trust in regulatory decisions.

Despite the valuable achievements towards a unified European view of clinical evidence, challenges remain. The need for ongoing adaptation to scientific advancements, such as personalized medicine and real-world evidence, necessitates continuous refinement of regulatory frameworks. Additionally, the integration of regulatory and HTA processes presents both opportunities and complexities. While regulatory agencies focus on benefit–risk assessments, HTA institutions evaluate therapeutic (added) value and cost-effectiveness. Closer collaboration and continuous intensive dialogue between these entities, not only on the European but on the international level, could improve decision-making frameworks, streamline decision-making, and optimize patient access to innovative treatments.

## 7. Conclusions

The harmonization of pharmaceutical regulations and the establishment of the centralized marketing authorization procedure has enabled efficient scientific collaboration and work-sharing, ensuring high-quality assessments and timely access to medicines in the EU. As the EU moves towards a more integrated HTA framework, lessons from regulatory harmonization—such as centralized coordination, standardized methodologies, and transparency—can support future developments. The integration of early dialogue, expert-driven assessments, and adaptive methodologies will be crucial in ensuring a harmonized yet flexible system that meets the diverse healthcare needs of member states. Many of these key findings, such as central coordination and patient/clinician engagement, to name just two examples, have already been well taken into account in the EU Regulation. Strengthening collaboration between regulatory and HTA bodies—from national to European to international levels—will be essential, too, in achieving a balanced approach that supports innovation while ensuring patient safety and economic sustainability.

## Data Availability

No new data were created or analyzed in this study. Data sharing is not applicable to this article.
